# Pediatric Vital Sign Distribution Derived From a Multi-Centered Emergency Department Database

**DOI:** 10.3389/fped.2018.00066

**Published:** 2018-03-23

**Authors:** Robert J. Sepanski, Sandip A. Godambe, Arno L. Zaritsky

**Affiliations:** ^1^Department of Quality Improvement, Children’s Hospital of The King’s Daughters, Norfolk, VA, United States; ^2^Department of Pediatrics, Eastern Virginia Medical School, Children’s Hospital of The King’s Daughters, Norfolk, VA, United States

**Keywords:** heart rate, respiratory rate, infant, child, emergency service, hospital

## Abstract

**Background:**

We hypothesized that current vital sign thresholds used in pediatric emergency department (ED) screening tools do not reflect observed vital signs in this population. We analyzed a large multi-centered database to develop heart rate (HR) and respiratory rate centile rankings and *z*-scores that could be incorporated into electronic health record ED screening tools and we compared our derived centiles to previously published centiles and Pediatric Advanced Life Support (PALS) vital sign thresholds.

**Methods:**

Initial HR and respiratory rate data entered into the Cerner™ electronic health record at 169 participating hospitals’ ED over 5 years (2009 through 2013) as part of routine care were analyzed. Analysis was restricted to non-admitted children (0 to <18 years). Centile curves and *z*-scores were developed using generalized additive models for location, scale, and shape. A split-sample validation using two-thirds of the sample was compared with the remaining one-third. Centile values were compared with results from previous studies and guidelines.

**Results:**

HR and RR centiles and *z*-scores were determined from ~1.2 million records. Empirical 95th centiles for HR and respiratory rate were higher than previously published results and both deviated from PALS guideline recommendations.

**Conclusion:**

Heart and respiratory rate centiles derived from a large real-world non-hospitalized ED pediatric population can inform the modification of electronic and paper-based screening tools to stratify children by the degree of deviation from normal for age rather than dichotomizing children into groups having “normal” versus “abnormal” vital signs. Furthermore, these centiles also may be useful in paper-based screening tools and bedside alarm limits for children in areas other than the ED and may establish improved alarm limits for bedside monitors.

## Introduction

Vital sign thresholds are incorporated into various screening tools to help identify those children at higher risk of serious medical or surgical illness (1–4). To effectively utilize vital sign data in children, however, it may be more useful to identify the magnitude of deviation from the expected vital sign distribution, considering the child’s age and location of care [e.g., emergency department (ED) versus intensive care unit (ICU)], rather than determining if the vital sign value is abnormal. Since most current scoring tools consider vital signs as dichotomous variables (i.e., “normal” or “abnormal”) within relatively wide age ranges, it is not surprising that most triage and scoring tools have performed poorly even though they are significantly associated with the outcome of interest (4–7).

Most screening tools use vital sign thresholds that fail to consider the physiologic stress response of a child seen in the ED. Thus, the upper “normal” vital sign thresholds observed in ED patients were higher than observed in children who were hospitalized on the ward or who were ambulatory (8–10). The value of using empirically derived ED vital sign thresholds was demonstrated in a study of a pediatric ED sepsis screening tool that incorporated temperature (TMP) adjustment for heart rate (HR) and respiratory rate (RR) (11). The tool’s positive predictive value was 48.7%, almost threefold better than using the consensus systemic inflammatory response syndrome (SIRS) criteria (12), with no loss of sensitivity (11).

In sepsis, early identification of children at risk is a key recommendation for optimal management (13) since early implementation of protocol-guided sepsis care decreased sepsis-related organ dysfunction, hospital and ICU length of stay, and mortality (14–16). Clinical judgment alone misses approximately 27% of septic children seen in the ED (17). Using vital sign data with current threshold parameters is limited by the high rate of tool activation; almost 17% of febrile or hypothermic children in the ED met alert criteria, but only 2.5% of these children had severe sepsis or septic shock (17). Similarly, more than 90% of febrile children in the ED meet vital sign criteria for SIRS (5), and ~12% of all ED children triggered an alert based on tachycardia alone (4). These data suggest that current sepsis screening tools identify too many at-risk children, leading to alert fatigue (18) and reluctance to use the tool.

Recent studies empirically derived centile ranks and, in some cases, *z*-scores for vital sign parameters by age (9, 11). The rationale for considering the vital sign parameter’s *z*-score or centile rank rather than “normal” versus “abnormal” is based on the enhanced statistical power of the former over the latter (19, 20).

We hypothesized that current pediatric HR and RR vital sign thresholds used in Pediatric Advanced Life Support (PALS) or derived from low-acuity ED patients do not accurately reflect empirically derived HR and RR centiles. To develop empirically derived thresholds that could be incorporated into ED screening tools and may inform monitor alarm limits, we analyzed a very large multi-institutional database to derive HR and RR centile ranks and *z*-scores stratified by age in children presenting to the ED. Ultimately, our goal is to derive HR and RR data that can be applied as continuous variables in electronic health record (EHR)-based tools to stratify children into risk groups or used as threshold limits in paper-based triage tools. Empirically derived vital sign distributions also may better determine alarm limits in different aged children to reduce alarm fatigue and may be useful to stratify children into risk groups for clinical trials.

## Materials and Methods

### Data Source

Data in Cerner Health Facts^®^ (21) are extracted directly from the EHR of hospitals in which Cerner has a data use agreement. All admissions, medication orders and dispensing, laboratory orders, and specimens are date and time stamped, providing a temporal relationship between treatment patterns and clinical information. Cerner Corporation has established Health Insurance Portability and Accountability Act-compliant operating policies to establish de-identification for Health Facts^®^.

### Data Analysis

**Figure 1:**
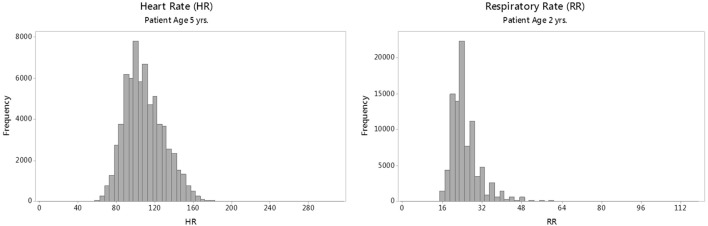
Sample raw distributions of individual heart rate (HR) and respiratory rate (RR) parameters for a single age group.

The HR and RR distributions by age were modeled using the generalized additive models for location, scale, and shape (GAMLSS) methodology and software (22, 23). GAMLSS adjusts for kurtosis and skews in the distributions and allows the generation of normalized standard centiles, or “*z*-scores,” and smooth centile curves by age. This process requires that data are fitted to one of several mathematical distributions (24) that approximate real-world distributions of vital sign measurements. For modeling HR, the Box–Cox power exponential (BCPE) distribution was chosen based on previous work (9, 25) and goodness of fit. For modeling RR, however, BCPE was unsuitable due to the highly leptokurtic (thin, slender peaked) and heavier tailed nature of the RR distributions (as shown in Figure 1); therefore, an alternative distribution, the Box–Cox “t” (BCT) (26), was employed.

For modeling the distributions of RR, a natural logarithm transformation was required for model convergence. It was also necessary to introduce Gaussian statistical noise of up to ±2 breaths/min (with a mean value of 0) to overcome the reduced variation due to digit bias in the raw RR measurements (9) and so induce greater conformity to the BCT modeling distribution.

For each vital sign, our modeling process entailed a stepwise fitting procedure to calculate optimal age-specific fits for mean, SD, skew, and kurtosis, with an additive term that used “penalized B-splines” to create smooth centile curves (22). The process was repeated with patient age raised to various exponents [designated in GAMLSS literature as the “power parameter” (27)] between 0.01 and 1 to further optimize model fit. The methodology used for smoothing is necessarily subjective in that the modeler may choose between iterative methods (22, 27) that result in varying degrees of over-fitting or under-fitting of the model to the empirical data. We chose the Schwarz Bayesian Criterion for minimizing local deviation between model and data, which resulted in acceptably smooth curves that retained a good fit to the underlying vital signs data (22). A technical specification of the GAMLSS parameters that describe our centile models is found in Data Sheet S1 in Supplementary Material.

Because our original intent was to examine both raw and TMP-corrected initial vital signs, our data capture was restricted to initial encounters having HR, RR, and TMP values taken within 15 min of one another. Encounters having two or more distinct measurements recorded for the same vital sign at the same date and time were not uncommon (comprising about 7% of the total). In such cases, we selected the average value, provided that the range of simultaneous values did not exceed 10% of the largest value for HR or RR (i.e., approximately 10–20 bpm for HR or 2–5 breaths/min for RR), or 3% of the largest value for TMP (i.e., approximately 1°C), arbitrarily chosen to exclude likely erroneous outliers; if exceeded, the encounter was excluded. Records in one or more of the following categories also were similarly excluded as likely outliers: (1) extreme (likely spurious) values of HR (<30 or >300 bpm), RR (0 or ≥120 breaths/min), or TMP (<30 or >46°C); (2) encounters classified as “Trauma Center” cases; or (3) encounters where the patient had a diagnosis of a chronic heart or respiratory condition present on admission. The latter two exclusion types (collectively ~0.4% of cases) were excluded since we did not want to include children who were more likely to have very abnormal vital signs. A summary of the selection and exclusion criteria used to determine the final data set for our study is given in Data Sheet S2 in Supplementary Material with details on specific diagnostic exclusions in Data Sheet S3 in Supplementary Material. A sensitivity analysis of the effects of these exclusions on the final modeled centile results was conducted, as described below.

### Model Validation

To test model reproducibility, we performed a stratified split-sample validation whereby the full data set used for each vital sign was divided into a “training”subset consisting of two-thirds of randomly selected records from each age group, and a “test” data subset consisting of the remaining one-third of records. The training subset was modeled by GAMLSS to generate centile cutoffs, and the percentage of records with vital sign values above and below these modeled cutoffs for the 95th, 99th, 5th, and 1st centiles, respectively, were compared between the training and test data subsets using exact chi-square tests. Holm (step-down Bonferroni) (28) corrections, performed separately for HR and RR, were used to adjust for the multiple testing of each metric.

### Sensitivity Analysis

To determine the effect of excluding encounters classified as “Trauma Center,” or those where the patient had a diagnosis of a chronic heart or respiratory condition, the modeling of HR and RR distributions was repeated including these encounters. The resultant modeled centile values, rounded to the nearest whole number for HR and RR, were then compared between datasets of children with and without exclusions.

### Comparison to Empirically Derived and Guideline-Based Vital Sign Thresholds

We graphically plotted the empirically derived 5th, 50th, and 95th centiles by age compared with the same centiles calculated by O’Leary et al. (10), derived from a large single-center ED population. We also show the upper and lower HR and RR thresholds for awake children recommended in the American Heart Association Pediatric Emergency Assessment, Recognition, and Stabilization, and PALS courses (29).

## Results

### Data Selection

The Health Facts^®^ data used comprises initial vital sign values and relative measurement times for HR and RR collected from ED encounters involving children (ages 0–17 years) at 169 U.S. hospitals (five were children’s hospitals) for calendar years 2009 through 2013. For all encounters, only patient types classified as “Emergency” were selected, which excludes children who were hospitalized from the ED since the Health Facts database does not specifically identify those admitted patients who entered the hospital *via* the ED. Patient age was recorded as an integer age in months for children <2 years or in years for older children. Because ages were categorical rather than continuous, age was converted to represent each category by its midpoint (e.g., 0–1 month becomes 0.5 months) for modeling purposes.

Our preliminary analysis of patient TMPs taken in the ED found that TMP distributions varied according to patient age and route of measurement, making generalized TMP corrections of HR and RR problematic. Therefore, we analyzed HR and RR without TMP correction.

### Characteristics of Study Subjects

A total of 1,203,042 encounters were used to study the distribution of initial HR by age, with slightly fewer (1,202,984) available for an analysis of RR. Patient information and encounters included in our study for each of the vital signs examined are presented in Tables 1 and 2 and Data Sheet S4 and S5 in Supplementary Material. As expected, the sample sizes for age categories representing children <2 years were smaller than those for children 2 years or older due to the shorter age interval (1 month versus 1 year) represented by these categories (Table 1). Table 2 lists information on patient demographics and length of stay for contributing encounters. Data Sheet S4 in Supplementary Material presents patient encounter frequencies according to the contributing hospitals’ demographics. Data Sheet S5 in Supplementary Material summarizes contributing patient encounters by principal diagnosis using Clinical Classifications Software (30) categories, an Agency for Healthcare Research and Quality methodology for condensing patient ICD-9 diagnostic data into clinically meaningful groups.

**Table 1:** Number of contributing encounters by patient age group.

Age description	HR, RR^a^, *N* (% of total)	Age description	HR, RR^a^, *N* (% of total)
<1 month	12,860 (1.1%)	2 to <3 years	93,406 (7.8)
1 to <2 months	13,016 (1.1%)	3 to <4 years	82,813 (6.9)
2 to <3 months	13,204 (1.1%)	4 to <5 years	74,328 (6.2)
3 to <4 months	11,255 (0.9%)	5 to <6 years	68,517 (5.7)
4 to <5 months	11,846 (1.0%)	6 to <7 years	59,316 (4.9)
5 to <6 months	12,317 (1.0%)	7 to <8 years	50,664 (4.2)
6 to <7 months	12,895 (1.1%)	8 to <9 years	46,448 (3.9)
7 to <8 months	13,354 (1.1%)	9 to <10 years	44,818 (3.7)
8 to <9 months	13,580 (1.1%)	10 to <11 years	44,093 (3.7)
9 to <10 months	13,349 (1.1%)	11 to <12 years	43,225 (3.6)
10 to <11 months	13,234 (1.1%)	12 to <13 years	42,813 (3.6)
11 to <12 months	13,002 (1.1%)	13 to <14 years	45,766 (3.8)
12 to <13 months	13,016 (1.1%)	14 to <15 years	50,007 (4.2)
13 to <14 months	12,078 (1.0%)	15 to <16 years	53,940 (4.5)
14 to <15 months	11,548 (1.0%)	16 to <17 years	59,621 (5.0)
15 to <16 months	11,115 (0.9%)	17 to <18 years	65,264 (5.4)
16 to <17 months	10,805 (0.9%)		
17 to <18 months	10,351 (0.9%)		
18 to <19 months	10,012 (0.8%)		
19 to <20 months	9,476 (0.8%)		
20 to <21 months	9,085 (0.8%)		
21 to <22 months	9,011 (0.7%)		
22 to <23 months	8,878 (0.7%)		
23 to <2 years	8,716 (0.7%)		

*^a^Overall numbers for respiratory rate (RR) are slightly less than for heart rate (HR) (1,202,984 versus 1,203,042), but percentages of total for each age group as rounded are identical for HR and RR*.

**Table 2:** Contributing encounters: patient demographics and length of stay.

Encounter characteristics	HR, RR^a^
	Mean/Median
Length of Stay (hours)	5.7/2.6
Gender	N (% of total)
Male	627,095 (52.1%)
Female	575,834 (47.9%)
Missing/Unknown/Other	113 (0.0%)
Race/ethnicity	
Caucasian	508,180 (42.2%)
African American	423,607 (35.2%)
Missing/Unknown/Other	140,270 (11.7%)
Hispanic	100,929 (8.4%)
Asian	17,524 (1.5%)
Native American	12,532 (1.0%)

Total	1,203,042^a^

*^a^Overall numbers for respiratory rate (RR) are slightly less than for heart rate (HR) (1,202,984 versus 1,203,042), but mean/median length of stay and percentages of total for each demographic characteristic as rounded are identical for HR and RR*.

Representative raw distributions (histograms) for the HR and RR (Figure 1) illustrate the positive skew (longer right tail) in the distributions of each vital sign, and the high kurtosis and heavy tails evident in the distribution of RR.

### Centile Curves

**Figure 2:**
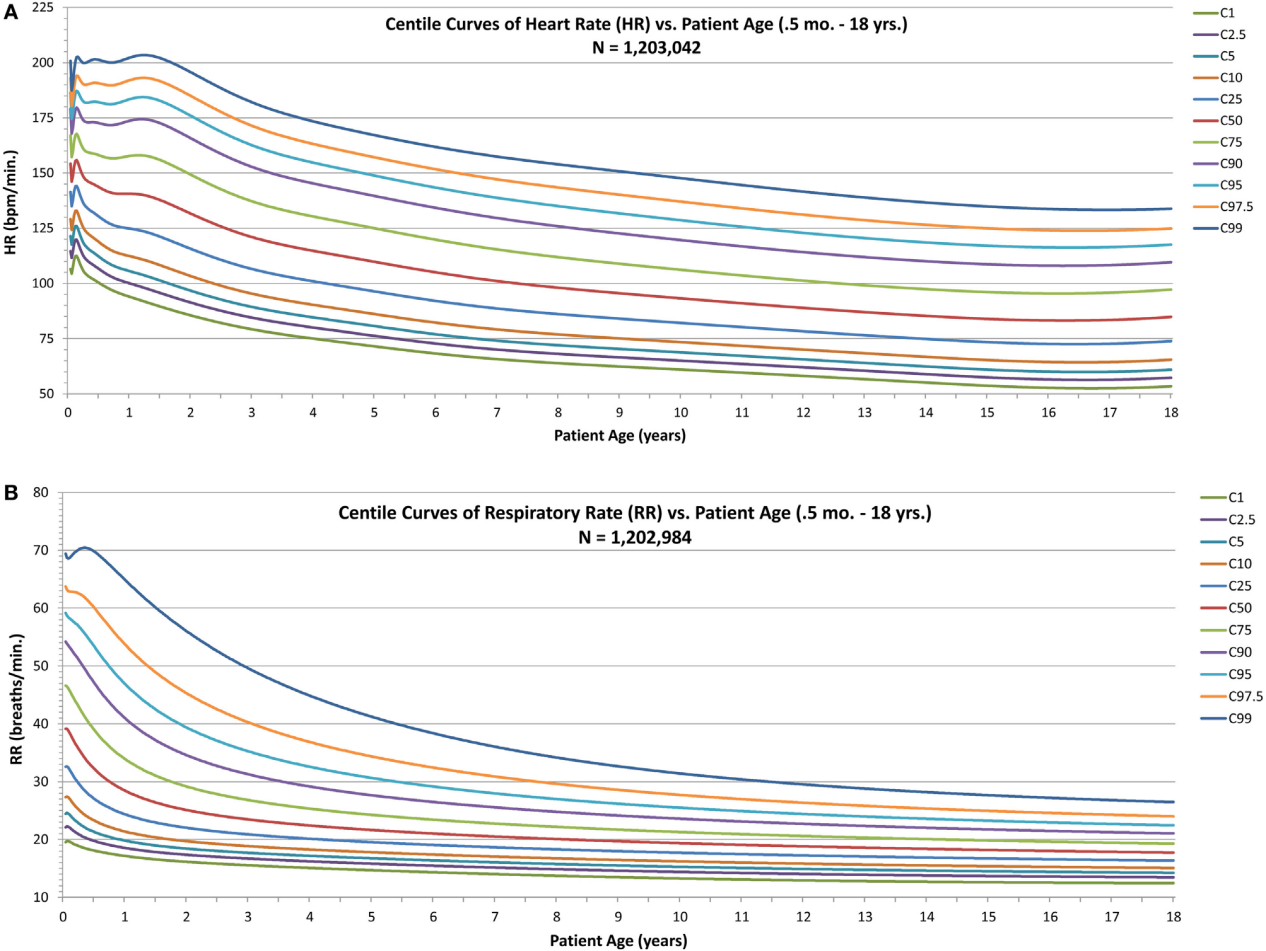
Modeled centile curves for pediatric vital sign metrics. **(A)** The centiles from the first centile (C1) to the 99th centile (C99) for heart rate (HR) by age and **(B)** the respiratory rate (RR) centiles.

For HR and RR (Figures 2A,B), the centile curves reveal a somewhat complex relationship of vital sign distributions with age for neonates and infants up to about two years of age, followed by smoothly decreasing vital sign values with age for all centiles, with values becoming nearly constant over the late adolescent age range.

### Model Validation

The results of the split-sample validation of model result reproducibility (Table 3) for all metrics and each centile group tested showed no significant difference in the proportion of cases identified by applying the model derived cutoffs obtained from the training data subset to the empirical data in the training and test subsets, respectively, based on either the raw or Holm-adjusted chi-square P statistic.

**Table 3:** Validation of vital sign centile modeling results.

Vital sign metric	Modeled^a^ centile group	Training set, *N*^b^ (%)	Test set, *N*^b^ (%)	Raw *P*^c^	Holm-adjusted *P*^d^
HR	>99th	6,653 (0.83)	3,325 (0.83)	0.99	1.0
HR	>95th	42,540 (5.30)	20,980 (5.23)	0.09	0.37
HR	<5th	36,890 (4.60)	18,477 (4.61)	0.85	1.0
HR	<1st	7,771 (0.97)	3,776 (0.94)	0.15	0.44
RR	>99th	7,862 (0.98)	3,961 (0.99)	0.70	0.86
RR	>95th	39,921 (4.98)	19,828 (4.94)	0.43	0.86
RR	<5th	31,091 (3.88)	15,834 (3.95)	0.06	0.22
RR	<1st	5,246 (0.65)	2,692 (0.67)	0.28	0.83

*^a^Using cutoffs obtained by modeling data in training set*.

*^b^Number of encounters with vital sign metric greater than (for 95th and 99th) or less than (for 5th and 1st) modeled cutoff*.

*^c^Comparison of cases above or below cutoff between training and test sets using exact chi-square (df = 1)*.

*^d^Holm (step-down Bonferroni) corrections, performed separately for heart rate (HR) and respiratory rate (RR) were employed to adjust for multiple testing (99th, 95th, 5th, and 1st centiles) of each metric*.

Table 3 also shows the high general agreement between the modeled centiles and the empirical data. Ideally, the modeled centile groups “>99th” and “<1st” should each identify about 1% of the empirical data, while groups “>95th” and “<5th” should each identify about 5%. Deviations between the modeled and the empirical percentages are apparent only in the RR “<5th” and “<1st” (which identify about 3.9 and 0.7% of the respective empirical data for these groups).

### Centile and *z*-Score Tables

The modeled values of HR, and RR from the 1st to 99th centiles for each age group are provided as Tables 4 and 5. Supplementary Material Data Sheet S6 and S7 in Supplementary Material present similarly formatted model results by age group for HR and RR as normalized standard centiles (*z*-scores) ranging from −3.0 to +3.0 SDs for HR and RR.

**Table 4:** Heart rate (HR, bpm) centiles^a^ by age.

Age (years) midpoint	Age/units midpoint	Age description	C1	C2.5	C5	C10	C25	C50	C75	C90	C95	C97.5	C99
0.042	0.5 months	<1 month	106	114	121	128	140	153	165	177	184	191	198
0.125	1.5 months	1 to <2 months	112	119	126	133	144	156	168	180	187	194	202
0.208	2.5 months	2 to < 3 months	108	115	121	128	139	151	163	176	184	192	201
0.292	3.5 months	3 to <4 months	105	111	117	124	135	147	160	173	182	190	200
0.375	4.5 months	4 to <5 months	102	109	115	121	133	145	159	172	181	190	200
0.458	5.5 months	5 to <6 months	101	107	113	120	131	144	158	173	182	190	201
0.542	6.5 months	6 to <7 months	100	106	111	118	130	143	158	173	182	191	201
0.625	7.5 months	7 to <8 months	98	104	110	117	128	142	157	172	182	190	201
0.708	8.5 months	8 to <9 months	97	103	109	115	127	141	157	172	182	190	201
0.792	9.5 months	9 to <10 months	96	102	107	114	126	141	157	172	182	190	201
0.875	10.5 months	10 to <11 months	95	101	106	113	125	140	157	172	182	191	201
0.958	11.5 months	11 to <12 months	94	100	106	112	125	140	157	173	183	191	201
1.042	12.5 months	12 to <13 months	93	100	105	112	124	140	158	174	183	192	202
1.125	13.5 months	13 to <14 months	93	99	105	112	124	140	158	174	184	193	203
1.208	14.5 months	14 to <15 months	92	98	104	111	124	140	158	174	184	193	204
1.292	15.5 months	15 to <16 months	92	98	103	110	123	140	158	174	184	193	204
1.375	16.5 months	16 to <17 months	91	97	103	110	123	139	157	174	184	193	203
1.458	17.5 months	17 to <18 months	90	96	102	109	122	138	157	173	183	192	203
1.542	18.5 months	18 to <19 months	89	95	101	108	121	137	156	172	182	191	202
1.625	19.5 months	19 to <20 months	89	95	100	107	120	136	154	171	181	190	201
1.708	20.5 months	20 to <21 months	88	94	99	106	119	135	153	170	180	189	200
1.792	21.5 months	21 to <22 months	87	93	98	105	118	134	152	169	179	188	198
1.875	22.5 months	22 to <23 months	86	92	98	104	117	133	151	167	178	187	197
1.958	23.5 months	23 months to <2 years	86	92	97	104	116	132	150	166	176	185	196
2.5	2.5 years	2 to <3 years	82	88	93	99	111	126	143	159	169	178	189
3.5	3.5 years	3 to <4 years	77	82	87	93	103	118	133	149	158	167	177
4.5	4.5 years	4 to <5 years	73	78	82	88	98	112	127	142	152	160	170
5.5	5.5 years	5 to <6 years	70	75	79	84	94	107	122	137	146	154	165
6.5	6.5 years	6 to <7 years	67	71	76	81	90	103	118	132	141	149	160
7.5	7.5 years	7 to <8 years	65	69	73	78	87	99	113	128	137	145	155
8.5	8.5 years	8 to <9 years	63	67	71	76	85	97	110	124	133	141	152
9.5	9.5 years	9 to <10 years	62	66	70	74	83	94	107	121	130	138	149
10.5	10.5 years	10 to <11 years	60	64	68	73	81	92	105	118	127	136	146
11.5	11.5 years	11 to <12 years	59	63	67	71	79	90	103	116	125	133	143
12.5	12.5 years	12 to <13 years	57	61	65	69	77	88	100	113	122	130	140
13.5	13.5 years	13 to <14 years	56	59	63	67	75	86	98	111	119	127	138
14.5	14.5 years	14 to <15 years	54	58	62	66	74	84	96	109	117	125	135
15.5	15.5 years	15 to <16 years	53	57	61	65	73	84	96	108	116	124	134
16.5	16.5 years	16 to <17 years	53	57	60	65	73	83	96	108	116	124	133
17.5	17.5 years	17 to <18 years	53	57	60	65	73	84	96	109	117	124	134

*^a^Centiles abbreviated as C1 (first centile) to C99 (99th centile)*.

### Sensitivity Analysis

Each sensitivity analysis compared modeled centiles between data sets with and without predefined exclusions for 11 centile levels and 40 age categories (as presented in Tables 4 and 5). Our finding of only one discrepancy out of 440 combinations of centile and age for HR and four for RR—with differences of just 1 bpm for HR and 1 breath/min for each RR—shows that our choice to exclude these encounters resulted in a negligible effect on modeled centile values compared with those obtained without the exclusions.

**Table 5:** Respiratory rate (RR, breaths/minute) centiles^a^ by age.

Age (years) midpoint	Age/units midpoint	Age description	C1	C2.5	C5	C10	C25	C50	C75	C90	C95	C97.5	C99
0.042	0.5 month	<1 month	19	22	24	27	33	39	47	54	59	64	69
0.125	1.5 months	1 to <2 month	20	22	24	27	32	38	45	53	58	63	69
0.208	2.5 months	2 to <3 month	19	21	23	26	30	37	44	52	58	63	70
0.292	3.5 months	3 to <4 month	19	21	23	25	29	35	42	51	57	63	71
0.375	4.5 months	4 to <5 month	18	20	22	24	28	34	41	49	56	62	71
0.458	5.5 months	5 to <6 month	18	20	21	23	27	33	40	48	54	61	71
0.542	6.5 months	6 to <7 month	18	20	21	23	27	32	38	46	53	60	70
0.625	7.5 months	7 to <8 month	18	19	21	23	26	31	37	45	51	58	69
0.708	8.5 months	8 to <9 month	18	19	20	22	26	30	36	44	50	57	67
0.792	9.5 months	9 to <10 month	17	19	20	22	25	30	35	43	49	56	66
0.875	10.5 months	10 to <11 month	17	19	20	22	25	29	35	42	48	55	65
0.958	11.5 months	11 to <12 month	17	19	20	21	24	29	34	41	47	54	64
1.042	12.5 months	12 to <13 month	17	18	20	21	24	28	34	40	46	53	64
1.125	13.5 months	13 to <14 month	17	18	20	21	24	28	33	40	46	52	63
1.208	14.5 months	14 to <15 month	17	18	20	21	24	28	33	39	45	51	62
1.292	15.5 months	15 to <16 month	17	18	19	21	24	27	32	39	44	51	62
1.375	16.5 months	16 to <17 month	17	18	19	21	23	27	32	38	44	50	61
1.458	17.5 months	17 to <18 month	17	18	19	21	23	27	32	38	43	50	61
1.542	18.5 months	18 to <19 month	17	18	19	20	23	27	31	37	43	49	60
1.625	19.5 months	19 to <20 month	17	18	19	20	23	26	31	37	42	48	59
1.708	20.5 months	20 to <21 month	16	18	19	20	23	26	30	36	41	48	59
1.792	21.5 months	21 to <22 month	16	18	19	20	23	26	30	36	41	47	58
1.875	22.5 months	22 to <23 month	16	18	19	20	22	26	30	35	40	47	58
1.958	23.5 months	23 month to <2 years	16	17	19	20	22	25	29	35	40	46	57
2.5	2.5 years	2 to <3 years	16	17	18	19	21	24	28	33	37	43	53
3.5	3.5 years	3 to <4 years	15	16	17	18	20	23	26	30	34	38	47
4.5	4.5 years	4 to <5 years	15	16	17	18	20	22	25	28	31	35	43
5.5	5.5 years	5 to <6 years	14	16	17	18	19	21	24	27	30	33	39
6.5	6.5 years	6 to <7 years	14	15	16	17	19	21	23	26	28	31	37
7.5	7.5 years	7 to <8 years	14	15	16	17	18	20	22	25	27	30	35
8.5	8.5 years	8 to <9 years	14	15	16	17	18	20	22	25	27	29	33
9.5	9.5 years	9 to <10 years	13	15	15	16	18	20	22	24	26	28	32
10.5	10.5 years	10 to <11 years	13	14	15	16	18	19	21	23	25	28	31
11.5	11.5 years	11 to <12 years	13	14	15	16	17	19	21	23	25	27	30
12.5	12.5 years	12 to <13 years	13	14	15	16	17	19	20	22	24	26	29
13.5	13.5 years	13 to <14 years	13	14	15	15	17	18	20	22	24	25	28
14.5	14.5 years	14 to <15 years	13	14	14	15	17	18	20	22	23	25	28
15.5	15.5 years	15 to <16 years	13	14	14	15	17	18	20	22	23	25	27
16.5	16.5 years	16 to <17 years	12	14	14	15	17	18	20	21	23	24	27
17.5	17.5 years	17 to <18 years	12	14	14	15	16	18	19	21	23	24	27

*^a^Centiles abbreviated as C1 (first centile) to C99 (99th centile)*.

### Comparison to Current and Guideline Vital Sign Threshold

**Figure 3:**
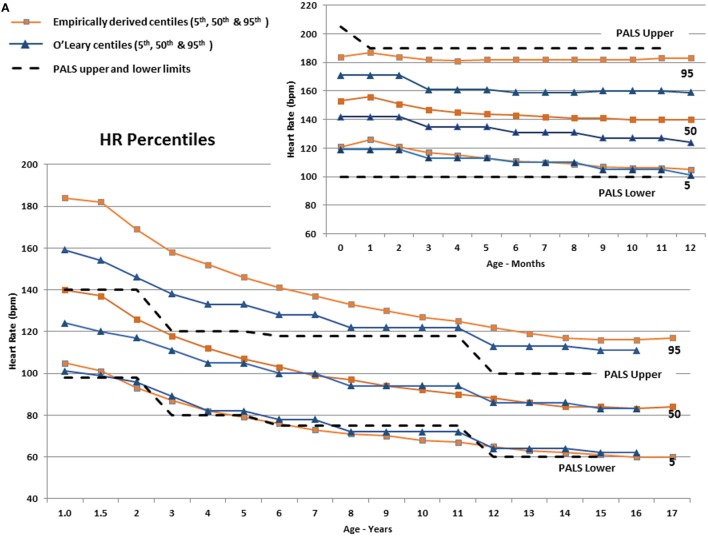

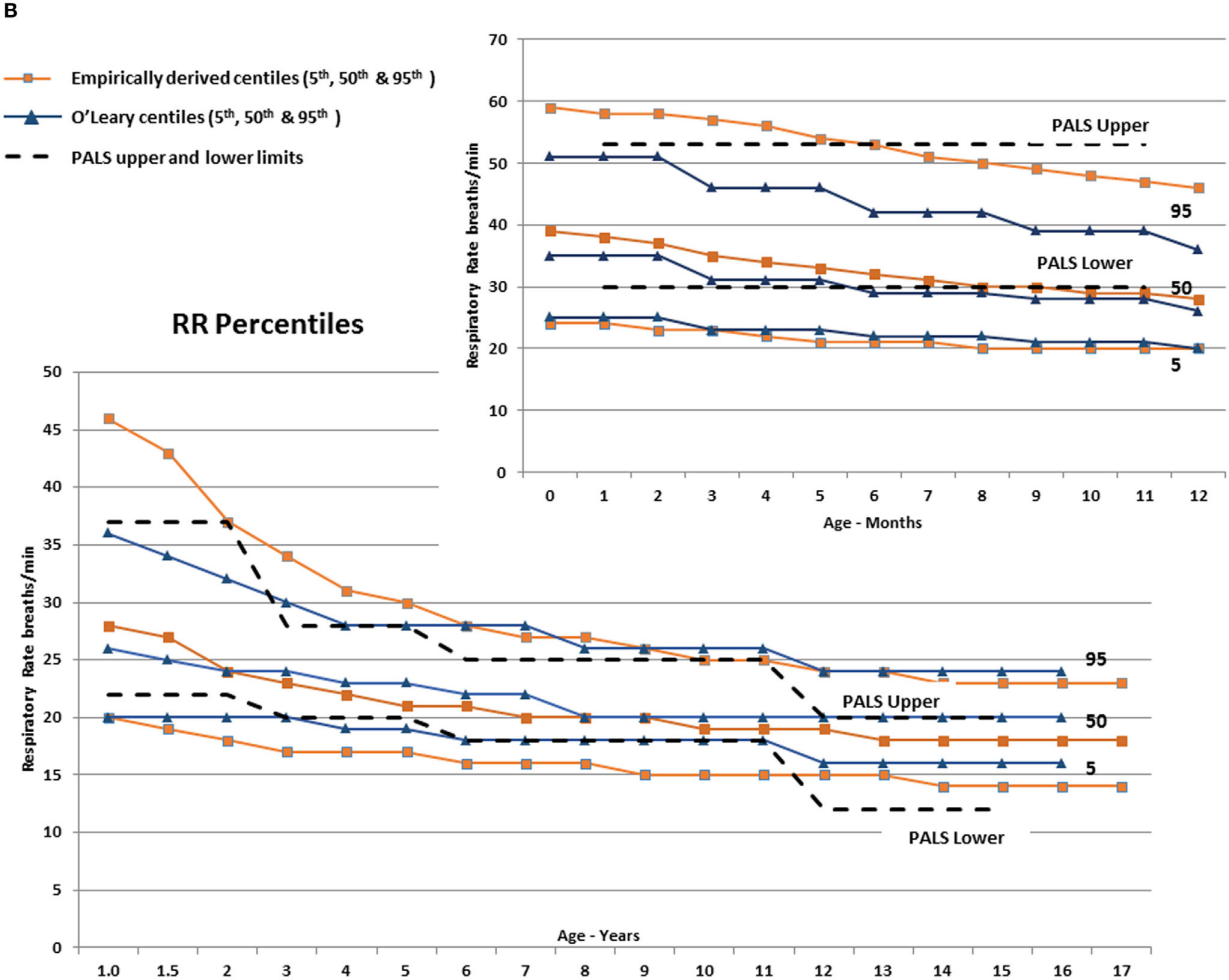
**(A)** The derived 5th, 50th, and 95th HR centiles by age compared with similar centiles derived by O’Leary et al. (10) The Pediatric Advanced Life Support (PALS) (29) upper and lower HR limits are also shown (dashed lines). The smaller panel shows the centiles from 0 to 12 months of age. **(B)** The derived 5th, 50th, and 95th RR centiles by age compared with similar centiles derived by O’Leary et al. (10). The PALS (29) upper and lower RR limits are also shown (dashed lines). The smaller panel shows the centiles from 0 to 12 months of age.

In infants, Figure 3A shows that the empirically derived 50th centile for HR was 9–16 bpm higher than the values determined by O’Leary et al. (10), and the 95th centiles for HR were 13–24 bpm higher than the O’Leary values, whereas the infant-aged PALS recommended upper and lower HR limits (29) were above and below the 95th and 5th centiles, respectively. The empirically derived 95th centile RR in infants (Figure 3B) was also higher by 7–11 breaths/min than O’Leary’s data, whereas the 50th and 5th centile were similar. The PALS recommended upper RR in infants was similar to the empirically derived 95th centile range, although by 10 months of age, it steadily exceeded the empirically derived 95th centile. The lower PALS RR limit was 6–10 breaths/min above the empirically derived 5th centile and was similar to the 50th centile by 7 months of age.

In children (≥1 year), Figure 3A shows substantial discrepancy between the empirically derived 95th centile HR and O’Leary’s (10) centile HR values up to around 10 years of age. The fifth centile for all three sets of data are similar, but the PALS upper HR limits are below the empirically derived 95th centile and are between the 50th and 75th centile for age up to ~6 years old (23–44 bpm lower from 1 through 6 years of age). Similarly, the empirically derived 95th centile RR (Figure 3B) is 3–6 breaths/min higher than the PALS and O’Leary values up to 4–5 years of age.

## Discussion

We hypothesized that empirically derived HR and RR distributions would deviate from PALS recommended distributions, and values derived from a low-acuity population of children seen in the ED. Ideally, an effective screening tool should balance high sensitivity to detect children at risk of deterioration while limiting too many false-positive patients leading to alert fatigue (5). It is important to recognize, however, that a vital sign-based screening tool is unlikely to identify all at-risk children. Instead, recent data show that combining an EHR-based screening tool with clinician identification, timelier joint team assessments and improved escalation of care processes results in high sensitivity and specificity for identification of severe sepsis/septic shock (31).

As seen in Figures 3A,B, the utility of “normal” vital sign thresholds recommended by PALS (29), APLS (32), and other reference texts is limited since they group normal values into relatively wide age ranges, which encompass large physiologic ranges and thus a wide expected distribution of vital signs, and they are not empirically derived. The PALS upper HR thresholds are well below the empirically derived 95th centiles and are between the 50th and 75th centile for age up to ~6 years, leading to substantial over-identification of at-risk children.

Similarly, the empirically derived 95th centile RR (Figure 3B) is higher than the PALS and O’Leary values up to 4–5 years of age. The upper PALS RR limit would over-identify many adolescents, whereas the lower PALS RR values generally exceed the empirically derived value up to 12 years of age falling 2–3 breaths/min below the empirically observed fifth centile. Using PALS criteria also would overclassify up to 50% of older infants as having an abnormally low RR (Figure 3B).

Thus, it is not surprising that in a ward inpatient sample of over 116,000 observations, 54% of the HR and 40% of the RR vital signs observed in hospitalized children were outside textbook “normal” vital sign distributions and 38% of HR and 30% of RR observations would have resulted in increased early warning scores (9). Similarly, in an analysis of 40,356 ED visits at Colorado Children’s Hospital (5), 16.3% of the children had a fever (>38.5°C) and 92.8% of these febrile children met SIRS vital sign criteria, as defined by the International Pediatric Sepsis Conference (12). Most of these children were discharged without any intervention.

Several groups have used data from EHR’s to redefine the distribution of vital signs observed in children in the ED (8, 10) or hospital wards (9, 33). These analyses clearly show that the standard textbook and guideline distributions and thresholds for “abnormal” for vital signs do not reflect empirically observed distributions (9). However, our empirically derived centiles differ from centiles derived from a large (111,696) data set of children presenting to a single ED (10). Of note, this study (10) restricted its analysis to the lowest acuity children, whereas our analysis included all acuities, but excluded children seen in the ED who were subsequently admitted. This may better represent the distributions of vital signs seen in children brought to the ED, who are likely stressed by the ED environment, but who presumably are not critically or seriously ill and, thus, do not require hospitalization.

We believe our empirically derived centiles based on a very large, multi-centered database of ED visits by children provide evidence-based vital sign parameters to use in either EHR or paper-based early warning scores, to set monitor alarm limits and to risk-stratify children for clinical trials or epidemiologic studies. Rather than using dichotomous threshold values, which are often set by consensus, to define “normal” from “abnormal,” we believe that these data can lead to the development of more useful objective risk stratification tools.

Although using vital sign parameter *z*-scores or centile ranks rather than “normal” versus “abnormal” is complex, EHR systems can be programmed to analyze these data to risk-stratify children, which enhances the statistical power of analyzing continuous data rather than using a dichotomous threshold (19, 20). By necessity, dichotomizing a continuous physiologic variable into two categories anticipates an unrealistic step function for risk at the threshold level. For example, if the upper limit of normal HR is set at 180 bpm, then an infant with a HR of 179 bpm is considered “normal,” even though this infant is not demonstrating the same physiologic response as the same aged infant with a HR of 120 bpm.

The potential value of using risk-stratified assessment of vital signs was seen in an ED-based study of 1,750 febrile children evaluated using seven different vital sign modeling strategies to identify children with serious bacterial infections (34). The model that worked best utilized the degree of deviation of age-adjusted HR and RR from median values, with or without TMP correction.

### Centile and *z*-Scores

Although we generated smooth curves for the individual vital signs, the raw vital sign distributions for RR shown in Figure 1 are not Gaussian but rather show a strong kurtosis and right-tailed skew. The skewed RR may represent an increased prevalence of disease processes (i.e., respiratory conditions), which lead to more frequent elevated RR values in children presenting to the ED compared with a population of healthy, normal children.

We performed a stratified split-sample validation that showed (Table 3) for each vital sign metric there was no significant difference in the proportion of cases in the training versus the test subjects.

### TMP-Adjusted Vital Signs

Most screening tools do not TMP adjust the HR or RR. It is well known that increased TMP increases metabolic demand, thus increasing HR and RR (33, 35, 36). Analysis of vital sign data in hospitalized children (not ICU) found a near linear increase in HR with increasing TMP of approximately 10 beats/min for each degree Centigrade increase in TMP, although the increase appears to vary by age, with the HR increasing by 15 bpm in infants per degree Centigrade versus 8 bpm in 14- to 18-year-olds (33).

We planned to analyze the effect of TMP on HR and RR, but we were not confident in adjusting oral TMP to core TMP and did not know how to correct for axillary or temporal artery TMPs. A recent systematic review of the accuracy of peripheral versus core TMPs in adults and children (37) found few studies comparing oral to core TMP in children. Axillary and infrared tympanic TMPs can vary widely from core TMP, especially in patients who have poor perfusion. Moreover, an inpatient study conducted at Cincinnati Children’s Hospital and Children’s Hospital of Philadelphia observed significantly different TMP histograms between the institutions, which disappeared when one of the institutions transitioned to the same thermometer used at the other hospital, showing that different thermometers can introduce additional variation in the measured TMP (33).

### Limitations

Since we did not have precise ages, we used 1-month ranges (if <2 years of age) or 1-year age ranges for older children. It seems likely that the large sample size and smoothing of the data will limit the impact of including children within an entire year, especially since within any year range there is sizable normal variation based on child size and height as seen with blood pressure (38).

We chose to use extreme exclusion criteria for HR and RR. This may have resulted in the inclusion of data from children with erroneous or very abnormal vital sign values, but again the very large data set likely limits the impact of these outliers.

Further studies are needed to determine if using empirically derived vital sign thresholds would result in a lower sensitivity to identify high-risk children. Our previous analysis using empirically derived higher vital sign thresholds based on data from a single ED noted improved positive predictive value for severe sepsis/septic shock identification without affecting sensitivity (11).

## Conclusion

In summary, the derived HR and RR centiles and *z*-scores from more than 1 million children seen in the ED of mostly adult hospitals were often different from currently derived centiles in children seen in the ED and from consensus-based PALS guideline vital sign thresholds. We believe our data provide a reliable representation of HR and RR distributions in stressed children seen in the ED who do not require hospitalization. These data could be used to create algorithms within EHR’s to develop pragmatic risk scores that increase sensitivity and specificity and reduce alarm fatigue characteristic of dichotomous vital sign thresholds. We also believe these parameters may help establish improved alarm limits for bedside monitors and these empirically derived HR and RR thresholds may improve the performance of paper-based tools, such as those based on PALS vital sign thresholds. Finally, the data may better inform the creation of disease-specific screening tools.

## Availability of Data and Material

The datasets used and analyzed during the current study are available from the corresponding author, but restrictions apply to the availability of these data since they were obtained through a written agreement with Cerner Corporation and so are not publicly available. Data may be available from the author on reasonable request and with permission of Cerner Corporation.

## Ethics Statement

Since we analyzed de-identified clinical data, this is not human subjects research.

## Author Contributions

RS, SG, and AZ each made substantial contributions to the conception and design of the study. RS conducted the analyses and derived the centile curves and *z*-scores. RS and AZ jointly drafted and all three authors critically revised the manuscript for important intellectual content. All authors read and approved the final manuscript.

## Conflict of Interest Statement

The authors declare that the research was conducted in the absence of any commercial or financial relationships that could be construed as a potential conflict of interest.

## Abbreviations

ED, Emergency Department; EHR, electronic health record; HR, heart rate; GAMLSS, generalized additive models for location, scale, and shape; RR, respiratory rate; TMP, temperature; BCPE, Box–Cox Power Exponential; BCT, Box–Cox “t”; PALS, Pediatric Advanced Life Support; PEARS, Pediatric Emergency Assessment, Recognition and Stabilization; CCS, Clinical Classifications Software; ICU, intensive care unit; C, Centile (percentile).
