# Supplementary material for: Pediatric Vital Sign Distribution Derived From a Multi-Centered Emergency Department Database
Source: Front Pediatr. 2018 Mar 23;6:66. doi: 10.3389/fped.2018.00066 (PMC5876311; doi:10.3389/fped.2018.00066)
Supplement: Supplementary file 2 [file Data_Sheet_2.docx]

Appendix 2. Number of Records Excluded from Final Data Set for HR and RR

| Description of Records and Exclusions | **N** | % of total |
| --- | --- | --- |
| Records Meeting Initial Vital Sign (VS) Criteria* | 1,209,433 | 100 |
| (A) Exclusion Due to Extreme VS Measurements† | 129 | 0.01 |
| (B) Exclusion Due to Multiple Simultaneous VS Measurements Having  Excessive Range‡ | 1,058 | 0.09 |
| (C) Exclusion Due to Classification as Trauma Center Admission | 3,885 | 0.3 |
| (D) Exclusion Due to Diagnosis of Chronic Heart Condition,  Present on ED presentation | 632 | 0.05 |
| (E) Exclusion Due to Diagnosis of Chronic Respiratory Condition,  Present on ED presentation | 708 | 0.06 |
| Total Exclusions (A, B, C, D or E)** | 6,391 | 0.5 |
| Records Included in Final Data Set | 1,203,042 | 99.5 |

* Initial HR, RR, and temperature (TMP) measurements taken within 15 minutes of one another

† HR < 30 or > 300 beats/min, RR=0 or ≥ 120 breaths/min, TMP < 30 °C or > 46 °C

‡ Simultaneous range > 10% of maximum value for HR or RR, or > 3% of maximum value for TMP

** Not mutually exclusive
