# Supplementary material for: Pediatric Vital Sign Distribution Derived From a Multi-Centered Emergency Department Database
Source: Front Pediatr. 2018 Mar 23;6:66. doi: 10.3389/fped.2018.00066 (PMC5876311; doi:10.3389/fped.2018.00066)
Supplement: Supplementary file 3 [file Data_Sheet_3.docx]

Appendix 3. Excluded Chronic Cardiovascular and Respiratory Conditions from Final Data Set*

| Exclusion Category | ICD-9 Code and Description of Diagnosis |
| --- | --- |
| Cardiovascular | 745.xx : Bulbus Cordis Anomalies and Anomalies of Cardiac Septal Closure (includes Common Truncus, Transposition of Great Vessels, Tetralogy of Fallot, Common Ventricle, Endocardial Cushion Defects, and Septal Defects of Atrium or Ventricle) |
| Cardiovascular | 746.xx : Other Congenital Anomalies of the Heart (includes Valve Anomalies, Atresia, and Stenosis; Ebstein’s Anomaly; Hypoplastic Left Heart Syndrome; Cor Triatriatum; Coronary Artery Anomaly, Congenital Heart Block; Congenital Cardiomegaly; and Malposition of the Heart) |
| Cardiovascular | 747.xx: Other Congenital Anomalies of the Circulatory System (includes Patent Ductus Arteriosus; Coarctation and Other Anomalies of the Aorta; and Anomalies the Pulmonary Artery, Great Veins, or Peripheral Vascular System) |
| Respiratory | 748.4, 748.5, 748.6x: Congenital Anomalies of the Respiratory System (includes Congenital Cystic Lung; Agenesis, Hypoplasia, and Dysplasia of the Lung; Congenital Bronchiectasis; and Other Anomalies of the Lung) |
| Respiratory | 277.0x: Cystic Fibrosis |
| Respiratory | 496: Chronic Airway Obstruction |
| Respiratory | 516.6x: Interstitial Lung Diseases of Childhood |
| Respiratory | 518.83: Chronic Respiratory Failure |
| Respiratory | 756.6: Anomalies of the Diaphragm |
| Respiratory | 770.2: Interstitial Emphysema (originating in the perinatal period) |

*Where recorded as present on ED entry
