# Supplementary material for: Pediatric Vital Sign Distribution Derived From a Multi-Centered Emergency Department Database
Source: Front Pediatr. 2018 Mar 23;6:66. doi: 10.3389/fped.2018.00066 (PMC5876311; doi:10.3389/fped.2018.00066)
Supplement: Supplementary file 4 [file Data_Sheet_4.docx]

Appendix 4. Number of Contributing Encounters by Characteristics of Contributing Hospital

| Hospital Characteristics | HR,RR*  N (% of total) |
| --- | --- |
| Number of Hospitals Contributing Data | 169 |
| Hospital Region (United States) |  |
| South | 533,142 (44.3%) |
| Midwest | 255,841 (21.3%) |
| Northeast | 213,887 (17.8%) |
| West | 200,172 (16.6%) |
| Hospital Bed Size |  |
| 500+ | 197,914 (16.5%) |
| 300-499 | 297,781 (24.8%) |
| 200-299 | 437,996 (36.4%) |
| 100-199 | 143,290 (11.9%) |
| 6-99 | 94,636 (7.9%) |
| < 5 | 31,425 (2.6%) |
| Hospital Urban/Rural Status |  |
| Urban | 1,188,504 (98.8%) |
| Rural | 14,538 (1.2%) |
| Hospital Is a Teaching Facility |  |
| Yes | 911,543 (75.8%) |
| No | 288,134 (24.0%) |
| Not Reported | 3,365 (0.3%) |
| Total | HR: 1,203,042 (100%) |

*Overall numbers for RR are slightly less than for HR (1,202,984 vs. 1,203,042), but percentages of total for each hospital characteristic as rounded are identical for HR and RR.
