# Supplementary material for: Pediatric Vital Sign Distribution Derived From a Multi-Centered Emergency Department Database
Source: Front Pediatr. 2018 Mar 23;6:66. doi: 10.3389/fped.2018.00066 (PMC5876311; doi:10.3389/fped.2018.00066)
Supplement: Supplementary file 5 [file Data_Sheet_5.docx]

Appendix 5. Most Common Principal Diagnosis among Contributing Encounters by Clinical Classification Software (CCS) Category^30^

| Principal Diagnosis Description  (CCS Category) | HR,RR  Freq. Rank  (% of total) |
| --- | --- |
| Diagnosis not available (Missing) | 1 (18.4%) |
| Other upper respiratory infections | 2 (10.2%) |
| Fever of unknown origin | 3 (4.6%) |
| Superficial injury, contusion | 4 (4.4%) |
| Otitis media and related conditions | 5 (4.0%) |
| Other injuries and conditions due to external causes | 6 (3.0%) |
| Sprains and strains | 7 (2.8%) |
| Abdominal pain | 8 (2.8%) |
| Open wounds of head, neck, and trunk | 9 (2.6%) |
| Asthma | 10 (2.4%) |
| Viral infection | 11 (2.4%) |
| Other lower respiratory disease | 12 (2.1%) |
| Nausea and vomiting | 13 (2.1%) |
| Allergic reactions | 14 (2.0%) |
| Noninfectious gastroenteritis | 15 (1.9%) |
| Acute bronchitis | 16 (1.7%) |
| Fracture of upper limb | 17 (1.6%) |
| Other gastrointestinal disorders | 18 (1.6%) |
| Skin and subcutaneous tissue infection | 19 (1.5%) |
| Open wounds of extremities | 20 (1.5%) |
| Intestinal infection | 21 (1.3%) |
| Other skin disorders | 22 (1.2%) |
| Inflammation, infection of eye | 23 (1.2%) |
| Urinary tract infections | 24 (1.1%) |
| Headache, including migraine | 25 (1.1%) |
| Pneumonia | 26 (1.0%) |
| Other upper respiratory disease | 27 (0.9%) |
| Other ear and sense organ disorders | 28 (0.8%) |
| Influenza | 29 (0.8%) |
| Epilepsy, convulsions | 30 (0.8%) |
| Other connective tissue disease | 31 (0.7%) |
| Intracranial injury | 32 (0.6%) |
| Nonspecific chest pain |  |
| Miscellaneous mental health conditions |  |
| Other non-traumatic joint disorders |  |
| ALL OTHER (224 Distinct Entries) | (14.9%) |
| ALL OTHER (210 Distinct Entries) |  |
| ALL OTHER (214 Distinct Entries) |  |
| Total | (100%) |
